# The Role of Primary Tumor Resection in Patients With Pancreatic Neuroendocrine Tumors With Liver Metastases

**DOI:** 10.3389/fonc.2022.838103

**Published:** 2022-03-08

**Authors:** Yu Mou, Zi-Yao Wang, Chun-Lu Tan, Yong-Hua Chen, Xu-Bao Liu, Neng-Wen Ke

**Affiliations:** Department of Pancreatic Surgery, West China Hospital, Sichuan University, Chengdu, China

**Keywords:** primary tumor resection, pancreatic neuroendocrine tumors, liver metastases, tumor differentiation, overall survival (OS)

## Abstract

**Background:**

Liver metastases (LMs) are common in advanced pancreatic neuroendocrine tumor (PNET) patients. Currently, the benefit of primary tumor resection (PTR) in the setting of PNET patients with liver metastases is still controversial in several guidelines.

**Methods:**

Data were extracted from the Surveillance, Epidemiology and End Results (SEER) database to evaluate this issue. The main index of interest in our study was overall survival time.

**Results:**

Information on 536 PNET patients with liver metastases from the SEER database was identified. A total of 214 patients (PTR group) received primary tumor resection, and more than half of them (132 patients) had synchronous LM resection. The other 322 PNET patients (non-PTR group) with liver metastases did not receive primary tumor resection. A significant survival benefit was gained from PTR when compared with non-PTR patients, both in OS (72.93 ± 2.7 *vs*. 36.80 ± 2.22 months) and 3- or 5-year survival rates (75.1% *vs*. 28.9% and 67.9% *vs*. 22.3%, respectively). No difference was found between PTR alone and PTR with synchronous LM resection. From univariate and multivariate analyses, younger age (<65 years) and good or moderate tumor differentiation may be more important when considering primary tumor resection. However, we found that all grades of tumor differentiation could result in a better overall survival time after primary tumor resection.

**Conclusion:**

Our study suggested that primary tumor resection in pancreatic neuroendocrine patients with liver metastases could result in a longer survival time. Primary tumor resection with synchronous liver metastasis resection was not related to a better survival benefit. This treatment strategy may routinely be taken into consideration in these patients.

## Introduction

Pancreatic neuroendocrine tumors (PNETs) are a heterogeneous group of neoplasms representing approximately 1% of all pancreatic cancers by incidence and 10% of pancreatic cancers by prevalence (1). Surgical resection remains the primary and potentially curative treatment approach for PNETs. However, most patients have metastatic disease at diagnosis that often occurs first in the liver, and approximately 28-77% of patients develop liver metastases (LM) in their lifetime (2, 3). Management of pancreatic neuroendocrine tumor liver metastases (PNELMs) may depend on whether the liver disease is resectable.

For patients with limited liver metastases, surgical resection of both the primary tumor and hepatic disease in a staged or synchronous fashion is recommended. The role and benefit of primary site resection (PTR) in patients with unresectable liver metastases are still controversial. A recent systematic review and meta-analysis showed that palliative resection of primary PNETs in patients with unresectable metastatic liver disease can increase overall survival time (OS), but there was a bias toward patients with better performance status, less advanced disease, or a tumor located in the body or tail of the pancreas (4). Similar findings were demonstrated in another meta-analysis, but the limitations of the included studies do not allow firm conclusions (5). Until now, there has been no adequate robust evidence for whether a primary tumor should be resected in the presence of unresectable liver metastases. Moreover, additional pancreatic resection morbidity, the relatively indolent behavior, and the lower symptomatic presentation of nonfunctional PNETs should be taken into consideration.

Therefore, we designed this study to investigate whether primary tumor resection has a survival benefit in patients with pancreatic neuroendocrine tumors with liver metastases, even if the liver metastases are unresectable.

## Materials and Methods

### Patient Collection and Data Source

We used SEER*Stat software version 8.3.8 to retrieve the data for our study from the Surveillance, Epidemiology, and End Research (SEER) database (SEER Research Data, 18 Registries, Nov 2019 Sub 2000–2017). The primary sites for tumors of the pancreas were based on the column of site and morphology, which was labeled C25.0 to C25.9. The patients were enrolled according to the International Classification of Disease for Oncology, third edition (ICD-O-3) histology/behavior codes: pancreatic endocrine tumor, malignant (8150/3), insulinoma, malignant (8152/3), glucagonoma, malignant (8153/3), vipoma, malignant (8155/3), somatostatinoma, malignant (8156/3), enterochromaffin-like cell tumor, malignant (8242/3), goblet cell carcinoid (8243/3), neuroendocrine carcinoma, NOS (8246/3), and atypical carcinoid tumor (8249/3). Patient demographics included sex, age at diagnosis, year of diagnosis, grade, tumor size, surgery for primary site (derived from column RX Summ—Surg Prim Site (1998+)), surgery for distant sites (derived from column RX Summ—Surg Oth Reg/Dis (2003+)), survival months, vital status and SEER cause-specific death classification.

The selection criteria were as follows: (1) patients who had one primary cancer only and pancreatic NETs was the first; and (2) patients who had liver metastasis only at the time of diagnosis without other known sites of metastasis. The exclusion criteria were as follows: (1) incomplete follow-up information; (2) unknown cause of death or death attributed to causes other than this cancer; and (3) unknown characteristics.

### Statistical Analysis

Statistical analysis was performed using IBM SPSS Statistics 21.0. Patients’ baseline characteristics, tumor characteristics, and treatments were compared by the Mann–Whitney *U* test or Pearson chi-squared test. Data are presented as percentages or mean values. We used Kaplan–Meier curves to analyze the overall survival time (OS), and the differences between groups were compared by the log-rank test. Univariate and multivariate analyses were performed using Cox proportional hazards models. Hazard ratios (HRs) and 95% confidence intervals (CIs) were calculated. A *p*-value less than 0.05 was defined as statistically significant.

## Results

### Baseline Characteristics of Patients and Tumors

**Figure 1:**
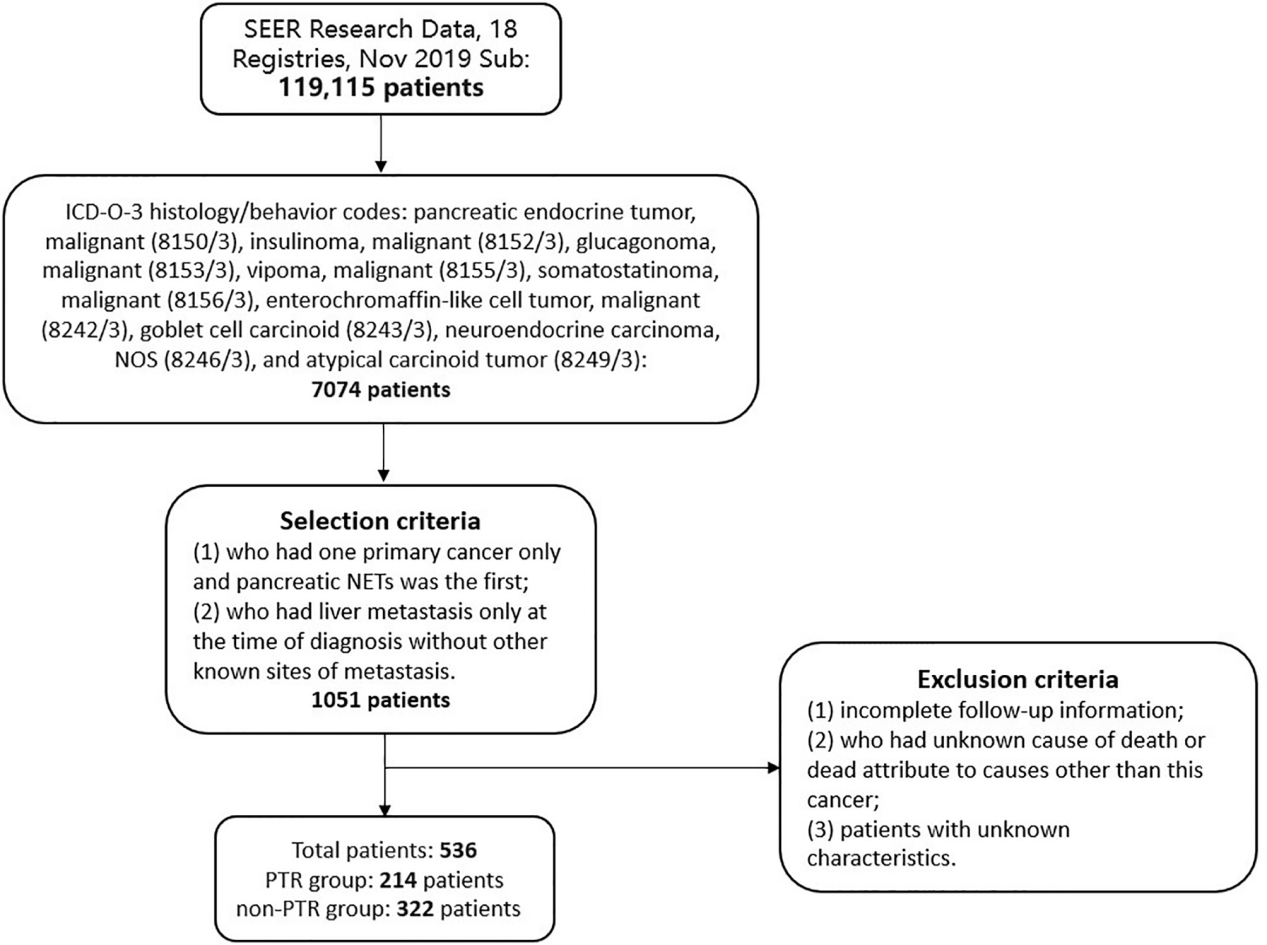
Flow chart of patients selection.

A total of 536 patients were included based on our inclusion criteria (**Figure 1**). All of these patients were diagnosed with pancreatic neuroendocrine tumors and had liver metastasis at diagnosis. The baseline characteristics are summarized in **Table 1**. There were 323 men and 213 women in this population, and the median age at diagnosis was 58 years. The number of patients with pancreatic head tumors (171 patients, 31.9%) was similar to that at the pancreatic tail (190 patients, 35.4%). The mean tumor size was 54.44 ± 31.57 mm, and 59.9% of tumors were more than 4 cm. All patients were pathologically diagnosed after resection surgery of biopsy, and the most common pathological type was neuroendocrine carcinoma, comprising 54.5% of these populations. Moreover, five tumors were functional PNETs, including two patients with insulinoma, two patients with gastrinoma, and one with glucagonoma. Based on the degree of differentiation, tumors were divided into four grades (grade I: well differentiated; grade II: moderately differentiated, grade III: poorly differentiated, grade IV: undifferentiated). Approximately 76.3% of patients were well and moderately differentiated. All patients had liver metastases at diagnosis without other known sites (such as lung, brain, bone) of metastases.

**Table 1:** Baseline characteristics of pancreatic neuroendocrine tumors with liver metastases.

	All patients (*n* = 536)	Primary tumor resection (*n* = 214)	Nonprimary tumor resection (*n* = 322)	*p*-value
Age	57.99 ± 13.77	60.61 ± 13.01	54.05 ± 13.87	0.000
Sex				0.073
Male	323 (60.3%)	119 (55.6%)	204 (63.4%)	
Female	213 (39.7%)	95 (44.4%)	118 (36.6%)	
Primary site				0.159
Head	171 (31.9%)	59 (27.6%)	112 (34.8%)	
Body	55 (10.3%)	23 (10.7%)	32 (9.9%)	
Tail	190 (35.4)	89 (41.6)	101 (31.4%)	
Neck	12 (2.2)	4 (1.9%)	8 (2.5%)	
Overlap lesions	60 (11.2%)	19 (8.9%)	41 (12.7%)	
NOS	48 (9.0%)	20 (9.3%)	28 (8.7%)	
Histology				0.047
Neuroendocrine carcinoma	292 (54.5%)	110 (51.4%)	182 (56.5%)	
Carcinoid tumor	172 (32.1%)	66 (30.9%)	106 (32.9%)	
Atypical carcinoid tumor	54 (10.1%)	27 (12.6%)	27 (8.5%)	
Neuroendocrine tumor	13 (2.4%)	9 (4.2%)	4 (1.2%)	
Insulinoma	2 (0.4%)	0 (0%)	2 (0.6%)	
Gastrinoma	2 (0.4%)	2 (0.9%)	0 (0%)	
Glucagonoma	1 (0.1%)	0 (0%)	1 (0.3%)	
Tumor differentiation				0.001
I. Well differentiation	248 (46.3%)	109 (51.0%)	139 (43.1%)	
II. Moderately differentiation	159 (29.7%)	73 (34.1%)	86 (26.7%)	
III. Poorly differentiation	98 (18.3%)	26 (12.1%)	72 (22.4%)	
IV. Undifferentiation	31 (5.7%)	6 (2.8%)	25 (7.8%)	
Tumor size (mm)	54.44 ± 31.57	52.41 ± 28.66	57.5 ± 35.34	0.337
≤2 cm	35 (6.5%)	14 (6.5%)	21 (6.5%)	
2–4 cm	180 (33.6%)	75 (35.1%)	105 (32.6%)	
>4 cm	321 (59.9%)	125 (58.4%)	196 (60.9%)	
Surgical procedure				
None		0	322	0.000
Partial pancreatectomy		104	0	
Pancreaduodenectomy		88	0	
Total pancreatectomy		22	0	

### Primary Tumor Resection and Survival Time

**Figure 2:**
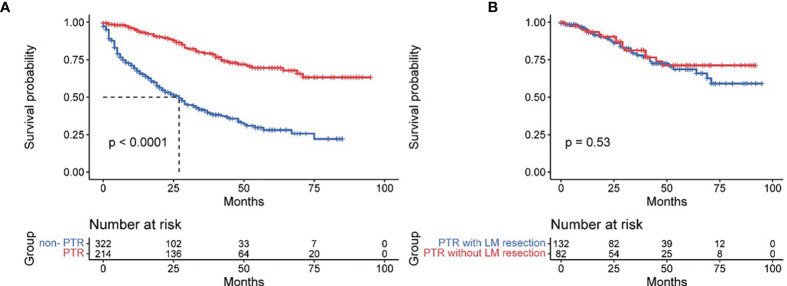
Effect of primary tumor resection **(A)** and with/without liver metastases resection **(B)** in pancreatic neuroendocrine tumor patients with liver metastases.

A total of 39.9% of patients (214 of 536 patients) received primary tumor resection, except for 8 patients who were recommended for surgery but not performed. The rest of the patients were not recommended for surgery. Surgical procedures included partial pancreatectomy (consisting of partial pancreatectomy and local excision of tumors), pancreaticoduodenectomy (with or without distal/partial gastrectomy), and total pancreatectomy. The median follow-up time was 43 months (1–95 months). The mean OS of all patients was 53.54 ± 2.03 months. Significant differences existed between PTR patients and non-PTR patients, and the OS of these two groups was 72.93 ± 2.70 and 36.80 ± 2.22 months, respectively (*p* = 0.000). The 3- and 5-year survival rates of PTR patients were 75.1% and 67.9%, respectively, while the same indexes of non-PTR patients were 28.9% and 22.3%, respectively. Additionally, we found no significant difference in the PTR group with or without LM resection. (PTR patients with LM resection vs. without LM resection: 71.48 ± 3.46 vs. 73.47 ± 4.01, *p* = 0.528) (**Figure 2**).

**Figure 3:**
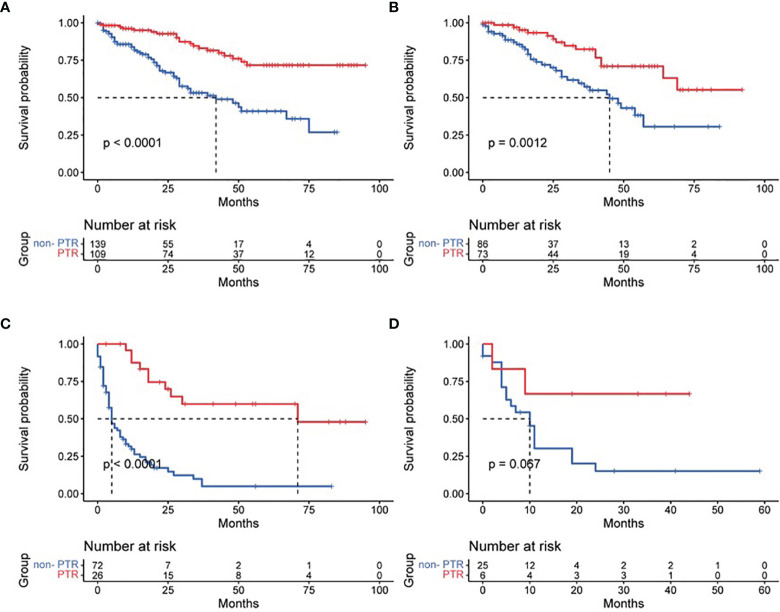
Effect of primary tumor resection in patients with different tumor differentiation: **(A)** well-differentiated patients; **(B)** moderately differentiated patients; **(C)** poorly differentiated patients; and **(D)** undifferentiated patients.

**Figure 4:**
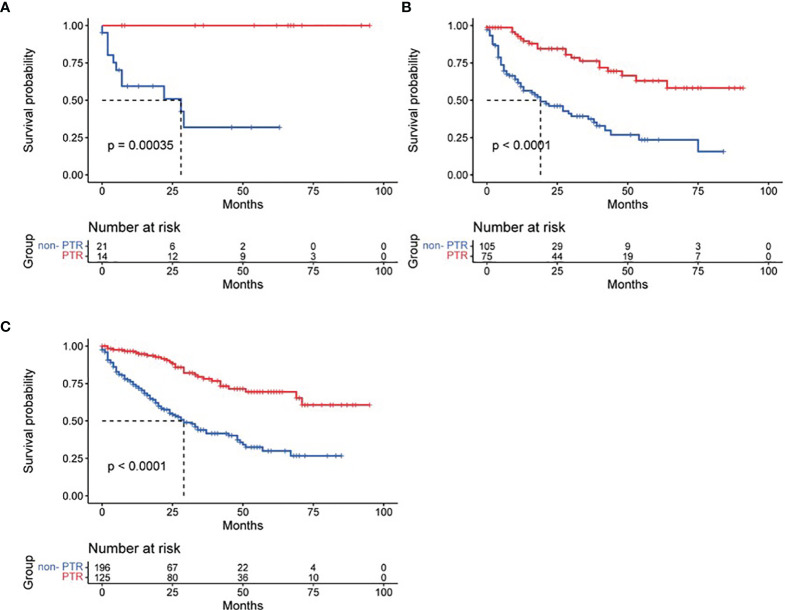
Effect of primary tumor resection in patients with different tumor sizes: **(A)** tumor size ≤2 cm; **(B)** tumor size 2–4 cm; **(C)** tumor size >4 cm.

**Figure 5:**
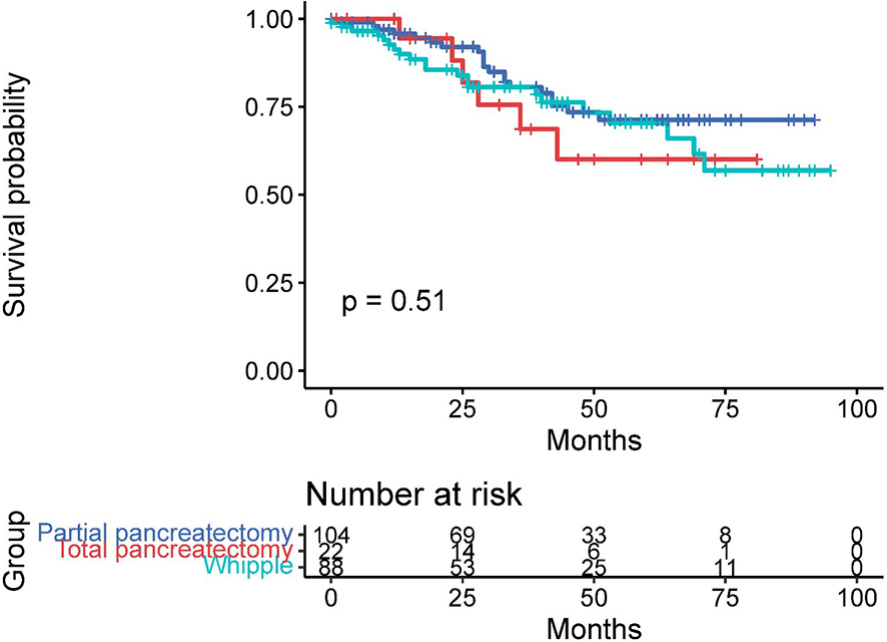
Overall survival time in patients who received different surgical procedures.

Based on tumor differentiation, all four grade groups showed that PTR significantly improved survival time (**Table 2**), especially in the grade III group (poor differentiation). The OS of the PTR patients was nearly 5-fold that of the non-PTR patients (64.58 ± 7.90 vs. 12.95 ± 2.53). In PTR patients, worse tumor differentiation was associated with decreased OS. The same results were shown when dividing patients based on tumor size into three groups (tumor size ≤2 cm, 2 cm < tumor size ≤4 cm, tumor size >4 cm). All 14 patients who received primary tumor resection with a tumor size less than 2 cm survived at the end of follow-up. Different surgical procedures also led to different outcomes. Patients who received partial pancreatectomy had better OS than the other two groups, which may be related to higher tumor differentiation, smaller tumor size, and lower additional mortality associated with the surgical procedure (**Figures 3**–**5**).

**Table 2:** Overall survival time in different groups.

	Primary tumor resection (*n* = 214)	Nonprimary tumor resection (*n* = 322)	*p*-value
Primary tumor resection	72.93 ± 2.70	36.80 ± 2.22	0.000
Liver metastases resection			
Yes	71.48 ± 3.46		
No	73.47 ± 4.01		
Tumor differentiation			
I: Well differentiation	77.250 ± 3.44	46.717 ± 3.49	0.000
II: Moderately differentiation	69.67 ± 4.71	46.5 ± 4.32	0.001
III: Poorly differentiation	61.58 ± 7.90	12.95 ± 2.53	0.000
IV: Undifferentiation	31.17 ± 7.46	16.31 ± 4.10	0.067
Tumor size (mm)			
≤2 cm	All alive	28.86 ± 6.41	
2–4 cm	66.37 ± 4.44	32.10 ± 3.68	0.000
>4 cm	72.45 ± 3.62	39.58 ± 2.86	0.000
Surgical procedure			
Partial pancreatectomy	74.24 ± 3.42		
Pancreaduodenectomy	70.72 ± 4.34		
Total pancreatectomy	60.35 ± 6.70		

From univariate and multivariate analyses, we found that age over 65 years (HR: 1.493, 95% CI: 1.137–1.962), poorly differentiated or undifferentiated tumors (HR: 4.102, 95% CI: 2.942-5.721; HR: 3.338, 95% CI: 2.043–5.455, respectively) and primary tumor resection (HR: 3.771, 95% CI: 2.702–5.263) were independent risk factors related to overall survival time (**Table 3**).

**Table 3:** Possible variables at univariate and multivariate analyses in pancreatic neuroendocrine patients with liver metastases.

Risk factors	Category	Univariate analysis		Multivariate analysis	
		Hazard ratio (95% CI)	*p*-value	Hazard ratio (95% CI)	*p*-value
Age	≤65 years	1		1	
	>65 years	1.73 (1.319–2.268)	0.000	1.493 (1.137–1.962)	0.004
Sex	Female	1			
	Male	0.869 (0.660–1.145)	0.319		
Primary tumor location	Head	1			
	Body	1.111 (0.664–1.860)	0.688		
	Tail	0.807 (0.420–1.553)	0.522		
	Neck	0.792 (0.472–1.329)	0.378		
	Overlapping lesions	0.879 (0.326–2.369)	0.799		
	NOS	0.986 (0.540–1.798)	0.963		
Tumor size	≤2 cm	1			
	2–4 cm	0.759 (0.409–1.407)	0.381		
	>4 cm	1.242 (0.937–1.647)	0.132		
Tumor differentiation	Well differentiation	1		1	
	Moderately differentiation	1.055 (0.734–1.517)	0.773	1.004 (0.698–1.445)	0.983
	Poorly differentiation	4.024 (2.895–5.595)	0.000	4.102 (2.942–5.721)	0.000
	Undifferentiation	4.093 (2.510–6.673)	0.000	3.338 (2.043–5.455)	0.000
Primary tumor resection	Yes	1		1	
	No	3.88 (2.800–5.396)	0.000	3.771 (2.702–5.263)	0.000
Surgical procedure	Partial pancreatectomy	1			
	Pancreaticoduodenectomy	0.651 (0.261–1.622)	0.357		
	Total pancreatectomy	0.881 (0.355–2.184)	0.784		

## Discussion

Current National Comprehensive Cancer Network (NCCN) guidelines for neuroendocrine tumors of the pancreas support resection of the primary site and metastases if complete resection is possible, and both staged and synchronous resection are recommended (6). However, the role of primary tumor resection for PNET patients with unresectable liver metastases is still controversial. For small intestinal neuroendocrine tumors (SI-NETs), palliative PTR may prevent or solve complications such as bowel obstruction or intestinal ischemia associated with primary tumors. Thus, primary tumor resection of intestinal NETs is strongly recommended even in the presence of liver or lymph node metastases (7). In contrast, a systematic review meta-analysis of midgut neuroendocrine tumor patients with unresectable metastatic liver disease suggested that PTR had a significant role in improving OS with a low perioperative risk of mortality (8). In the setting of unresectable PNELM, neither the European Neuroendocrine Tumor Society (ENETS) nor the North American Neuroendocrine Tumor Society (NANETS) guidelines recommend routine palliative primary resection (9, 10).

Our findings show that primary tumor resection in pancreatic neuroendocrine tumor patients with liver metastases is significantly associated with improved survival time. Furthermore, we evaluated potential risk factors related to OS. We found that age less than 65 years and well-differentiated or moderately differentiated tumor grade were associated with prolonged survival. Younger age and well-differentiated tumors may be important selected factors when considering primary tumor resection. Younger patients may have a better physical status to tolerate more aggressive treatment and fewer comorbidities. Citterio reported improved survival times in primary tumors resected from well-differentiated pancreatic NETs (median survival times were 138 and 37 months, respectively) (11). Furthermore, according to our findings, all differentiation grades had significantly better OS after PTR, especially in patients with poorly differentiated tumors, and PTR increased survival by nearly 5-fold.

Other factors, such as sex, tumor location, tumor size, and surgical procedures, were not significantly independent factors. Both the ENATS and NANETS guidelines proposed tumor location as a surgical selection factor. For nonfunctional PNETs, primary tumors located in the head of the pancreas are related to higher odds of specific symptoms, such as jaundice or duodenal occlusion, and these complications could be solved by endoscopic or surgical bypasses (9). In addition, distal pancreatectomy has lower morbidity than pancreaticoduodenectomy (Whipple procedure). Thus, primary lesions located in the body or tail may be more favorable for resection and derive better quality of life and outcomes (9, 10). A previous study supported the positive survival benefit of PTR in PNET patients of the body and tail with unresectable liver metastases when compared with non-PTR individuals (median survival time: 111 vs. 52 months) (12). We evaluated whether different surgical procedures had different outcomes, and the results showed that partial pancreatectomy and pancreaticoduodenectomy had similar OS. Moreover, univariate analysis also showed that both tumor location and surgical procedure were not significant independent risk factors. Tumor location and surgical procedures may not be limiting conditions in deciding whether to perform primary tumor resection with liver metastases.

Peptide receptor radionuclide therapy (PRRT) in somatostatin-positive NETs is a replacement treatment strategy in patients who are not suitable for radical resection, and PRRT could result in disease stabilization, partial remission, or reduction of tumor mass (13). A lower tumor burden and smaller lesions may allow a high dose of concentration and a higher chance of tumor response (14); thus, palliative or debulking surgery may increase the response to PRRT. Based on this hypothesis, a previous study in the setting of G1-G2 PNETs with diffuse liver metastases suggested that PTR prior to PRRT results in better progression-free survival (PFS) (70 vs. 30 months, *p* = 0.02) and OS (112 vs. 65 months, *p* = 0.011) (15). Another recent study also found that PTR before PRRT provides a significant survival benefit in patients with stage IV neuroendocrine neoplasms, and both PFS and OS improved (134 vs. 67 months, *p* < 0.001 and 18 vs. 14 months, *p* = 0.012, respectively) (16). These results provide us with a novel strategy for the combination of primary tumor resection and PRRT for advanced pancreatic neuroendocrine tumors with distant metastases.

Due to the relatively low incidence and heterogeneity of pancreatic neuroendocrine tumors, it is difficult to design randomized trials to provide strong evidence for standard treatment strategies. Our study also had some limitations due to its retrospective nature and selection bias. First, the tumor differentiation grade from the SEER database is different from the current guidelines, which are based on mitoses in a high power field and the Ki-67 index. Second, we do not have information about adjuvant therapies and postoperative therapies, which may influence the survival analysis in all patients. Third, the tumor burden of liver metastases (tumor location and number of lesions) may be a confounding variable. Fourth, the SEER database did not include tumor margin information.

Although several limitations exist in our study, we still suggest the significant role of primary tumor resection in pancreatic neuroendocrine tumors with liver metastases for improving survival time. Although all patients who receive resectable primary tumors may be potentially beneficial, younger patients and well- or moderately differentiated primary PNETs should be preferentially considered.

## Conclusions

Primary tumor resection is associated with longer survival in pancreatic neuroendocrine tumor patients with liver metastases, but additional synchronous liver metastasis resection was not related to better overall survival time. The combination of primary tumor resection and other treatment strategies (e.g., peptide receptor radionuclide therapy) may result in a better outcome.

## Data Availability Statement

The original contributions presented in the study are included in the article/supplementary material. Further inquiries can be directed to the corresponding authors.

## Author Contributions

N-WK and X-BL designed the study. Y-HC and C-LT acquired the data. Z-YW analyzed and interpreted the data. YM wrote the paper. N-WK critically revised the manuscript for important intellectual content. All authors listed have made a substantial, direct, and intellectual contribution to the work and approved it for publication.

## Funding

This research was supported by the Sichuan Province Science and Technology Planning Project (2020YFS0262), West China Hospital Clinical Research Incubation Project (21HXFH058), and the 1.3.5 Project for Disciplines of Excellence-Clinical Research Incubation Project (ZY2017302 and ZYJC21037), West China Hospital, Sichuan University.

## Conflict of Interest

The authors declare that the research was conducted in the absence of any commercial or financial relationships that could be construed as a potential conflict of interest.

## Publisher’s Note

All claims expressed in this article are solely those of the authors and do not necessarily represent those of their affiliated organizations, or those of the publisher, the editors and the reviewers. Any product that may be evaluated in this article, or claim that may be made by its manufacturer, is not guaranteed or endorsed by the publisher.

## Abbreviations

LM, liver metastases; PNETs, pancreatic neuroendocrine tumors; PTR, primary tumor resection; SEER, Surveillance, Epidemiology and End Results; PNELM, pancreatic neuroendocrine tumors liver metastases; OS, overall survival; NCCN, National Comprehensive Cancer Network; SI-NETs, small intestinal neuroendocrine tumors; ENETS, European Neuroendocrine Tumor Society; NANETS, North American Neuroendocrine Tumor Society; PRRT, peptide receptor radionuclide therapy; PFS, progression-free survival.
